# Validation of an LC-MS/MS method to determine five immunosuppressants with deuterated internal standards including MPA

**DOI:** 10.1186/1472-6904-12-2

**Published:** 2012-01-11

**Authors:** Armin Buchwald, Karl Winkler, Thomas Epting

**Affiliations:** 1 Division of Clinical Chemistry, Department of Medicine, University Medical Center Freiburg, Hugstetterstrasse 55, 79106 Freiburg, Germany

## Abstract

**Background:**

Therapeutic drug monitoring of immunosuppressive drugs in organ-transplanted patients is crucial to prevent intoxication or transplant rejection due to inadequate dosage. The commonly used immunoassays have been gradually undergoing replacement by mass spectrometry, since this physical method offers both a higher sensitivity and specificity. However, a switch should be carefully considered because it is a challenging procedure and needs to be thoroughly validated.

From an economic perspective it is reasonable to include mycophenolic acid into the assay, because this saves the necessity for an additional measurement. However, to date very few validation protocols for the measurement of immunosuppressants, including mycophenolic acid, are available. In order to adequately compensate for matrix effects, the use of stable isotope labeled internal standards is advisable. Here, the authors describe a single method suitable for the quantification of cyclosporine A, tacrolimus, sirolimus, everolimus and mycophenolic acid, based on deuterated internal standards.

**Methods:**

Plasma proteins were precipitated with zinc-sulfate, followed by an online solid phase extraction in the flow-through direction. Chromatographic separation was performed by a c18-phenyl-hexyl column. For subsequent mass spectrometric analysis stable-isotope-labeled internal standards were used. Results were available after 3.5 minutes.

**Results:**

Low quantification limits (accuracy: 104 - 118%) and linearity resulted in 2 -1250 ng/ml for cyclosporine A; 0.5 - 42.2 ng/ml for tacrolimus; 0.6 - 49.2 ng/ml for sirolimus; 0.5 - 40.8 ng/ml for everolimus and 0.01 - 7.5 μg/ml for mycophenolic acid. Intra-assay precision revealed a coefficient of variation (CV) of 0.9 - 14.7%, with an accuracy of 89 - 138%. The CV of inter-assay precision was 2.5 - 12.5%, with an accuracy of 90 - 113%. Recovery ranged from 76.6 to 84%. Matrix effects were well compensated by deuterated internal standards.

**Conclusions:**

The authors present a fast, economical and robust method for routine therapeutic drug monitoring comprising five immunosuppressants including mycophenolic acid.

## Background

Therapeutic drug monitoring (TDM) of immunosuppressive drugs in organ-transplanted patients is vitally important to prevent intoxication or rejection due to incorrect dosage. New therapeutic regimens combine immunosuppressants with different intracellular targets to lower blood concentrations and prevent undesired side effects [1-3]. This practice requires a precise and accurate analytical method, especially for the lower ranges of concentrations. With regards to sensitivity immunoassays often fail to meet clinical needs, due to their restricted detection limits. Moreover, they are vulnerable to cross-reactions against pharmacologically inactive metabolites, resulting in limited specificity and possibly false results [4].

In order to minimize imprecision at low drug concentrations, elaborate sample preparation is required to separate the molecules of interest from the patient's blood matrix molecules [5]. Any remaining matrix can adversely affect the efficiency of ionization and lead to erroneous results. Thus, matrix effects need to be identified and compensated by internal standards (IS). Since stable isotope labeled, structurally analogous isoforms are the most appropriate controls for matrix compensation [6,7], deuterated equivalents are about to replace the common IS ascomycin, cyclosporine D (CSD) [8-10] and carboxy butoxy ether of mycophenolic acid (MPAC).

Cyclosporine A (CSA), tacrolimus (TAC), everolimus (EVE) and sirolimus (SIR) are measured in whole blood, whereas mycophenolic acid (MPA) is determined in plasma [11,12]. Several methods have been reported to measure these drugs using different techniques for sample preparation and high pressure liquid chromatographic (HPLC) schedules [8,13-15]. However, these applications lack either appropriate IS or MPA to complete the analytical spectrum. Moreover, the separation of the main MPA metabolite mycophenolic acid glucuronide (MPAG) is essential for mass spectrometric analysis because MPAG can undergo in-source fragmentation to MPA via loss of the glucuronic acid moiety [11], which in the case of coelution is determined as MPA.

Generally, sample preparation consists of precipitation with a mixture of zinc-sulfate, organic solvent (methanol, acetonitrile or acetone) and IS, usually CSD, ascomycin and MPAC. The method proposed by Koster et al. for example precipitates proteins using zn-sulfate only for CSA and TAC, but not for SIR and EVE [16]. This approach has the disadvantage of requiring separate runs to get all of the analytes quantified.

Adding water before precipitation prevents sample clotting and improves extraction efficiency [17]. However, due to higher dilution this procedure needs a highly sensitive mass spectrometer.

One step that most sample preparation protocols described in the literature have in common is that after precipitation debris and contaminants must be removed, before the extracts undergo testing.

This necessitates two-dimensional chromatography with a 6 or 10 port switching valve. First, the sample is injected into the extraction column with aqueous extraction buffer containing a low concentration of organic solvent, after which it is then flushed at high flow rates (up to 5 ml/min). After valve switching, the organic buffer usually is run in back-flush mode to elute the analytes to the analytical column.

Another frequently used method is solid phase extraction in offline mode, which resembles the online procedure. The main difference is the desiccation of the eluted analytes under an airstream, followed by reconstitution in analytical buffer, before the sample can be injected into the HPLC-device.

This paper presents the complete validation of a single LC-MS/MS method for five immunosuppressants, including MPA, based on protocols described by Annesley et al. [17] and Seger et al. [15]. CYA, TAC, SIR and EVE were analyzed simultaneously in a single analytical run, whereas MPA was analyzed separately due to its preparation from plasma. To our knowledge this is the first application using corresponding deuterated IS (CSA-d4, TAC-^13^C-d2, EVE-d4, SIR-^13^C-d3, and MPA-d4) for each immunosuppressant and identical HPLC running conditions for all analytes. The use of an online solid-phase extraction in straight flush mode makes this application suitable for the determination of MPA as well.

## Methods

The protocol described in this paper follows internationally accepted guidelines (NCCLS, FDA) for the validation of in-house methods [18,19].

Patient material was used in anonymous form in consent with the ethics committee of the University Medical Faculty, Freiburg. According to the guidelines of ICMJE and WHO a study registration was not required.

### Reagents and materials

Immunosuppressants CSA, TAC, SIR and MPA, as well as water, methanol, acetic acid and ammonium acetate in LC-MS quality were purchased from Sigma-Aldrich (Munich, Germany). EVE was kindly provided by Novartis (Basel, Switzerland). MPAC was a kind gift of Roche Palo Alto, California. Zinc sulfate heptahydrate was supplied by Merck (Darmstadt, Germany).

Deuterated standards CSA-d_4_, TAC-^13^C-d_2_, EVE-d_4 _and MPA-d_3 _were obtained from Toronto Research Chemicals (Ontario, Canada) and SIR-^13^C-d_3 _from Alsachim (France). For calibration and quality control the 6Plus1 multilevel calibration kit and 4-level whole-blood controls of Chromsystems (Munich, Germany) were used. Calibrator and controls for MPA were provided by Recipe (Munich, Germany).

### Sample preparation

The concentrations of IS were adjusted to the area under the curve (AUC) of calibrator 2 (MPA: control 2) and were 50 μg/ml for CSA-d_4_, 0.8 μg/ml for TAC-^13^C-d_2_, 0.8 μg/ml for SIR-^13^C-d_3_, 0.25 μg/ml for EVE-d_4 _and 6 μg/ml for MPA-d_3_.

The working solution was prepared fresh daily and consisted of 30 ml methanol, 15 ml zinc sulfate solution (0.1 M) and 100 μl IS-Mix - sufficient for the treatment of 120 samples.

Samples were prepared according to the protocol described by Annesley et al. [17] and Seger et al. [15] with modifications as follows: 50 μl EDTA whole blood were taken for each sample to determine CSA, TAC, SIR and EVE, but 50 μl EDTA plasma was used to analyze MPA. In the instances of patients treated with a combination therapy including MPA, an aliquot of the EDTA sample was taken for plasma extraction just before the preparation of whole blood for CYA, TAC, SIR or EVE analysis. The latter were analyzed simultaneously in a single analytical run. Therefore, the plasma and whole blood preparations had to be injected separately. However, apart from different starting material, all preparation steps and analytical conditions were identical.

Samples, controls and calibrators were first mixed on a roller, hemolysed by adding 125 μl of water, thoroughly vortexed and then incubated for two minutes at room temperature (RT). The next step was an ultrasonic bath for 20 sec (omitted in the case of MPA). Initially, time dependent progression of hemolysis was confirmed by microscopy: immediately after the addition of water; then after 2 minutes of incubation with water and finally after 2 minutes incubation with water plus 20 sec of ultrasonic treatment. After the addition of 375 μl working solution samples were vortexed, kept for 10 minutes at RT and centrifuged at 15000 *g *(10 min). The supernatants were subsequently transferred to autosampler vials. The described procedure corresponds to a final dilution of 1:11 (v/v). To clarify whether matrix effects can be affected by a different dilution ratio, dilutions of 1:3 and 1:21 were carried out in parallel.

### Liquid chromatography

**Figure 1:**
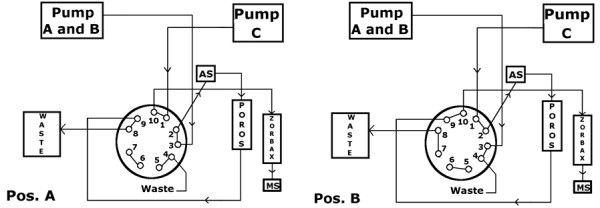
**Column Switching**. Pos A: Conditioning of the analytical column and loading of SPE column with aqueous buffer containing 10% methanol. Pos B: Elution from the analytical column (97% methanolic solvent) and rinsing step of precolumn with pure methanol.

For chromatographic separation, a Shimadzu Prominence HPLC system equipped with three isocratic pumps was used. The injection volume was 2 μl for MPA and 20 μl for the remaining immunosuppressive drugs. For online purification a solid phase extraction column (SPE) Poros R1/20, 2.1 mmD × 30 mm, 20 μm (Applied Biosystems, Darmstadt, Germany) was chosen. A phenyl-hexyl reversed phase C18 column Zorbax Eclipse XDB (Agilent, Waghäusel, Germany) 3.0 × 75 mm, 3.5 μm at 60°C was used as the analytical column. Column switching was controlled by a multi-channel valve (Valco 10 port 2 position valve) according to the pattern shown in Figure 1.

After injection, the SPE column was loaded for one minute with aqueous eluent (90:10 v/v, water/methanol containing 10 mM ammonium acetate and 0.1% acetic acid) at a flow rate of 5.0 ml/min. After valve switching organic molecules were eluted to the analytical column with methanolic buffer (97:3 v/v, methanol/water, 10 mM ammonium acetate and 0.1% acetic acid) at a flow rate of 0.9 ml/min. During that time both columns were connected in series, so that the flow direction corresponded to the loading direction. After switching the valve to the starting position, the Poros column was rinsed with pure methanol at 1 ml/min, to remove any remaining organic molecules and prevent carry-over contamination.

A 40 sec conditioning phase of the SPE column with aqueous buffer at a flow rate of 5 ml/min completed the run to a total analysis time of 3.5 min.

### Mass spectrometry

Mass spectrometric analysis was run on an API 4000 (AB Sciex, Darmstadt, Germany) equipped with an electrospray ionization interface (ESI). Analyte-specific voltage settings and ion-source adjustment were set manually, the former after automatic optimization. Gas settings in ml/min were: Collision gas: 7, curtain gas: 20, ion source gas 1: 50, ion source gas 2: 75, ion spray voltage: 5500 V, and temperature: 400°C. Ammonium adducts of each analyte were detected in positive ion mode by multiple-reaction-monitoring. Mass transitions can be found in Table 1. There is no qualifier for MPAG since this metabolite was not quantified but only monitored.

**Table 1:** Mass transitions of analytes and internal standards

Internal Standard Quantifier	Q1	Q3	Analyte Quantifier	Q1	Q3
Cyclosporine A-d_4_	1224.0	1206.9	Cyclosporine A	1219.9	1203.0

Tacrolimus^13^C-d_2_	824.6	771.6	Tacrolimus	821.5	768.4

Sirolimus-^13^C-d_3_	935.6	864.6	Sirolimus	931.6	864.7

Everolimus-d_4_	979.6	912.6	Everolimus	975.6	908.5

Mycophenolic acid-d_3_	341.2	210.1	Mycophenolic acid	338.2	207.1

Mycophenolic acid glucuronide	513.6	207.2			

Carboxy butoxy ether of mycophenolic acid	438.2	303.0			

**Internal Standard** **Qualifier**	**Q1**	**Q3**	**Analyte** **Qualifier**	**Q1**	**Q3**

Cyclosporine A-d_4_	1224.0	1188.8	Cyclosporine A	1219.9	1185.0

Tacrolimus^13^C-d_2_	824.6	789.4	Tacrolimus	821.5	786.5

Sirolimus-^13^C-d_3_	935.6	882.4	Sirolimus	931.6	882.4

Everolimus-d_4_	979.6	930.5	Everolimus	975.6	926.6

Mycophenolic acid-d_3_	341.2	306.3	Mycophenolic acid	338.2	210.1

Carboxy butoxy ether of mycophenolic acid	438.2	195.0			

### Validation methods

In order to determine the limit of quantification (LOQ) blank calibrator was spiked with low calibrator to obtain the desired concentration. All calibrators and controls were reconstituted according to the manufacturer's instructions, aliquoted and then frozen at -20°C. The LOQ was defined as the lowest measurable concentration which could be determined with a CV below 10%. For this, drug free blood was spiked with calibrator 1 (lowest concentration) to a concentration of 2 ng/ml for CSA, 0.5 ng/ml for TAC, 0.65 ng/ml for SIR and 0.55 ng/ml for EVE. The final concentrations varied due to a calibrator-saving common preparation step.

For MPA, plasma calibrator was prepared with drug-free plasma to a final concentration of 0.01 μg/ml. Aliquots of all three stock solutions were frozen.

Samples for the detection of linearity were prepared in drug-free blood pooled with calibrator 6 (highest concentration) at three different ratios (1:2, 1:4 and 4:1), to obtain a graph consisting of five data points. Native calibrator level 6 and blank blood served as starting points. In the case of MPA, drug-free plasma was spiked with mycophenolate to a concentration of 7.5 μg/ml and prepared in the same way. Final concentrations were as follows: CSA (blank; 224; 448; 672 and 896 ng/ml); TAC (blank; 10.6; 21.1; 31.7 and 42.2 ng/ml); SIR (blank; 12.3; 24.6; 36.9 and 49.2 ng/ml); EVE (blank; 10.2; 20.4; 30.6 and 40.8 ng/ml); MPA (blank; 1.88; 3.75; 5.63 and 7.50 μg/ml).

All specimens were aliquoted and stored at -20°C. To match the higher C2-values (blood concentration two hours after application), CSA dilutions were carried out with spiked whole blood up to 2000 ng/ml, because the highest calibrator level reaches just 896 ng/ml.

Precision tests were performed with drug-free whole blood samples from patients attending different clinical departments (internal medicine, surgery and gynecology). Enrichment with methanolic stock solutions of each drug yielded three ascending concentrations (lowest concentration = LOQ concentration). Due to the large volume required (3 ml for each drug and concentration add up to approx. 50 ml in total), the assays were not performed with certified controls. Furthermore, the use of the patient material came closest to the clinical environment. After gentle mixing for four hours on a roller mixer, samples were aliquoted and stored at -20°C until use.

For the intra-assay precision test, each concentration was extracted five times and measured in series.

The same specimens were used to detect the inter-assay precision on twenty continuous days (measured once), according to a new daily calibration.

To detect interference due to coeluted matrix constituents, the postcolumn infusion method as published by Taylor and Vogeser was implemented [7,20]. For this purpose methanolic solutions containing the immunosuppressants (500 ng/ml) were infused, while injecting analyte-free extracts of whole blood samples. Methanolic drug dilution was supplied at a constant flow rate of 10 μl/min to the HPLC flow of 0.9 ml/min with a T-piece directly before the ESI source, resulting in a further dilution of 1:90. After injecting drug-free blood matrix or methanol, total ion count (TIC) of the particular drug was monitored during the entire analytical cycle.

Process efficiency and recovery were tested by means of addition at pre- and post-extraction steps. For each analyte before sample preparation, drug free blood was spiked with three concentrations of methanolic stock solutions (CSA: 10, 100, 500 ng/ml, TAC, SIR, EVE: 2, 5, 10 ng/ml, and MPA: 0.5, 1, 5 μg/ml). The results were related to extracts that were enriched with corresponding methanolic stock solutions.

The stability of the extracted samples was checked by repeated determination after five hours (four patient samples were used for each analyte). The samples were kept cool throughout this time period in the autosampler.

An integral element of further quality assurance was the continuous participation in the international proficiency-testing scheme (Prof. Holt, London). For CSA, TAC and SIR three samples were supplied each month, for EVE three samples six times per year and for MPA two samples four times per year. Each scheme included samples which were either spiked to a known concentration or were pooled from patients receiving the target drug. Aliquots of these samples were extracted and measured on a daily basis for five consecutive days.

## Results

**Figure 2:**
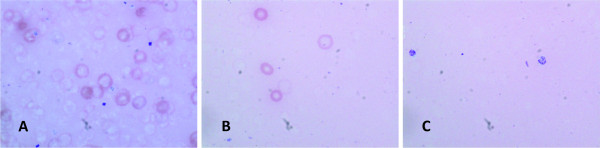
**Microscopic images of whole blood (50 μl) incubated with water (125 μl) for various times**. A: immediately after adding water B: 2 min incubation with water C: 2 min incubation with water plus 20 sec of ultrasonic treatment.

At the beginning of this validation, erythrocyte lysis was confirmed microscopically. In later tests it was checked only on a random basis. Ultrasonic treatment was introduced as a result of an inacceptable CV (> 15%) for inter-assay precision at the beginning of the validation process. Without ultrasonic treatment lysis was incomplete, so that numerous red blood cells remained undamaged. However, the ultrasound treated samples were lysed completely (Figure 2).

The advantage of stable-isotope-labeled isoforms for use as IS was shown by the variant ionization behavior of analytes and the corresponding deuterated standards compared to CSD and ascomycin in two different lots of certified control materials. In this case the signal for CSA and CSA-d4 increased to a similar extent (by 100%) compared to the previous lot, resulting in a correct calculation of the target value. In contrast, the signal for CSD rose only by half, leading to a false ratio and overestimation of the CSA target value.

The patients' samples were also measured in parallel using CSD and CSA-d4 as IS. In some cases the values calculated with CSD differed by 60% - 150% from the results obtained with CSA-d4. Comparable results could be obtained for ascomycin and TAC-^13^C-d_2_, EVE-d_4 _and SIR-^13^C-d_3 _respectively_. _Due to the experience with the certified control material and its known target values, further use of CSD and ascomycin was stopped.

A summary of validation results is depicted in Table 2. All drugs revealed linear behavior up to the highest concentration of calibrator. Assay ranges were 2 - 1250 ng/ml for CSA; 0.5 - 42.2 ng/ml for TAC; 0.6 - 49.2 ng/ml for SIR; 0.5 - 40.8 ng/ml for EVE and 0.01 - 7.5 μg/ml for MPA. The coefficient of determination (r^2^) was at a minimum of 0.997. Assay sensitivity as well as data for precision and accuracy exceeded clinical requirements, as defined as a minimum sensitivity of at least 1 ng/ml for TAC, SIR and EVE, 10 ng/ml for CSA and 0.02 μg/ml for MPA, and a CV for precision and accuracy below 20%. Surprisingly, the CV for LOQ concentrations was lower than for intra-assay precision.

**Table 2:** Validation results

Analyte	Measuring Range (ng/ml) MPA: μg/ml n = 3	LOQ, CV (%) Accuracy (%) n = 5	**Sample Conc**. (ng/ml) MPA: μg/ml	Intra Assay Precision (%) n = 5	Inter Assay Precision (%) n = 20	Accuracy (%) Intra/Inter Assay
Cyclosporine A	2 - 1250	3.7	2	7.4	7.1	125/113
	r^2^= 0.997	104	100	2.3	2.9	104/106
			500	0.9	2.5	106/105

Tacrolimus	0.5 - 42.2	7.5	0.5	12.3	9.1	138/100
	r^2^= 0.998	108	10	3.7	4.9	116/99
			20	3.8	4.5	111/98

Sirolimus	0.62 - 49.2	4.6	1	12.0	11.5	125/107
	r^2^= 0.999	106	10	5.1	5.0	100/102
			20	5.0	4.9	108/102

Everolimus	0.53 - 40.8	6.4	1	14.7	12.5	89/90
	r^2^= 0.999	118	10	2.9	6.9	97/96
			20	5.1	5.7	102/97

MPA	0.01 - 7.5	7.0	0.02	11.0	9.8	97/102
	r^2^= 0.999	105	0.2	1.3	3.2	97/97
			5	1.6	3.0	102/99

(LOQ: low limit of quantification, CV: variation coefficient)

The calibration results are summarized in Table 3.

**Table 3:** Calibration results (Intra Assay Precision)

Analyte	Target Value (ng/ml) MPA (μg/ml)	Measured Value (ng/ml) MPA (μg/ml)	Accuracy (%)	Linear Regression (1/x weighting)
Cyclosporine A	0	< 0	N/A	y = 0.00419 × + 0.000559
	23.5	24.5	104	r = 0.9996
	127	121	94.9	
	299	291	97.3	
	484	506	105	
	703	714	102	
	896	877	97.8	

Tacrolimus	0	< 0	N/A	y = 0.145 × + 0.00678
	2.1	2.45	117	r = 0.9994
	5.8	6.06	105	
	12.1	12.3	102	
	18.1	18.7	103	
	24.4	24.2	99.3	
	42.4	41.1	97.0	

Sirolimus	0	< 0	N/A	y = 0.104 × + 0.0171
	2.6	2.25	86.7	r = 0.9996
	6.6	6.21	94.2	
	12.8	13.0	102	
	20.0	20.8	104	
	29.0	28.9	99.5	
	49.2	49.1	99.7	
				

Everolimus	0	< 0	N/A	y = 0.112 × + 9.76e-0.09
	2.2	2.25	102	r = 0.9992
	5.9	5.66	95.8	
	11.6	11.2	96.3	
	17.0	18.0	106	
	23.2	24.0	103	
	40.8	39.6	97	

MPA	4.27	1-point calibration linear through zero		y = 0.00494 x r = 1.0

Recovery after sample preparation resulted in a process efficiency of 84 ± 0.6% for TAC, 80.7 ± 1.4% for EVE, 80.2 ± 1.3% for CSA, 77.1 ± 2.6% for SIR and 76.6 ± 1.9% for MPA.

**Figure 3:**
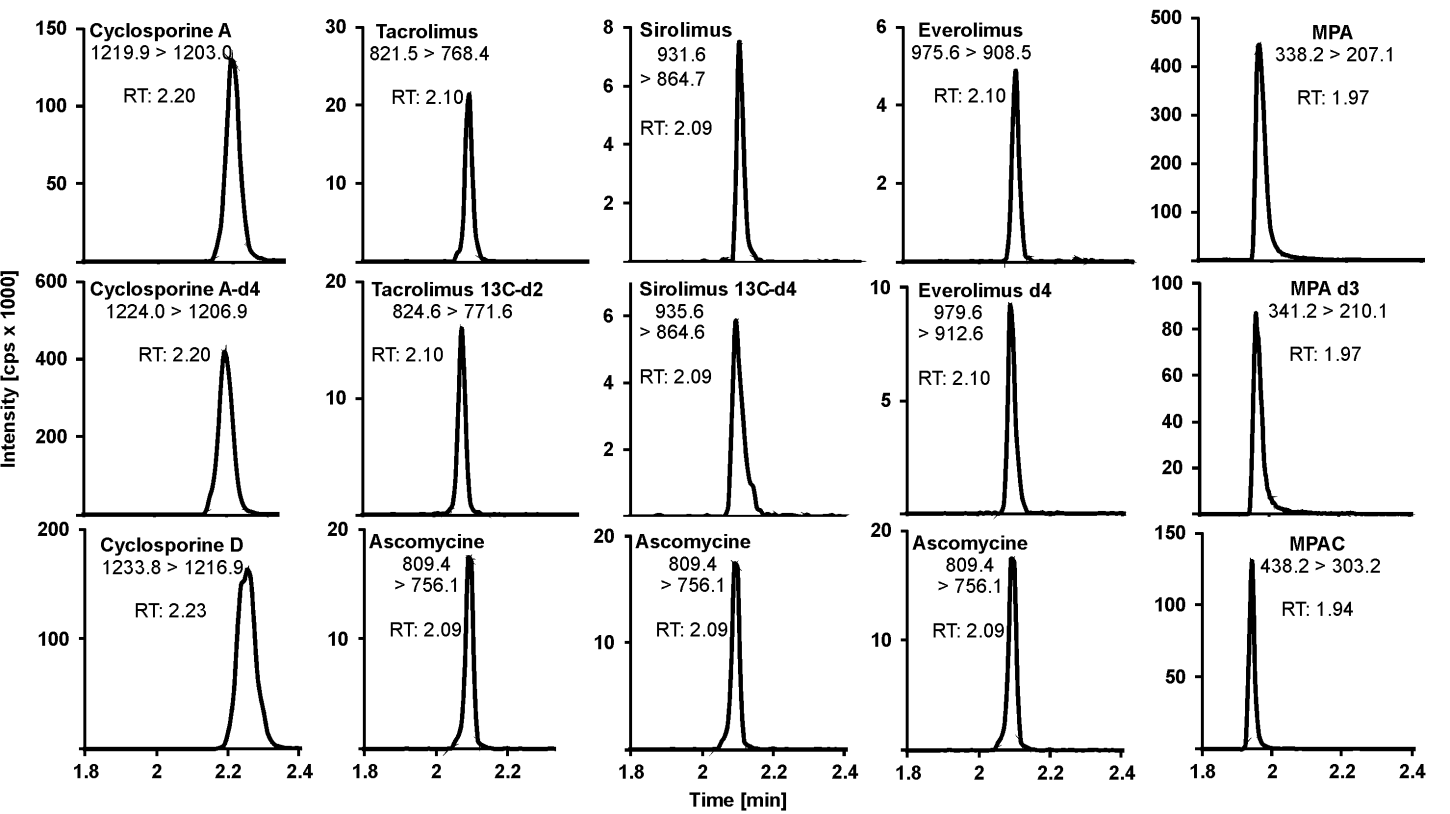
**Exemplary chromatograms of original routine patient samples containing 103 ng/ml CSA, 9.6 ng/ml TAC, 6.1 ng/ml SIR, 5.3 ng/ml EVE and 4.9 μg/ml MPA**. Mass transitions are stated.

Figure 3 shows exemple chromatograms of patient samples for each analyte and their corresponding IS. Additionally, common IS are compared with deuterated IS. All peaks exhibit a nearly symmetrical shape; retention of analytes and deuterated standards are concordant in time, whereas the common standards show a slight difference in retention time.

The additional rinsing of the SPE column with methanol for one minute, during separation in the analytical column, doubled the column lifetime to at least three months or 4500 analyses. The analytical quality, reflected by the shape of the peaks, remained stable throughout this time.

**Figure 4:**
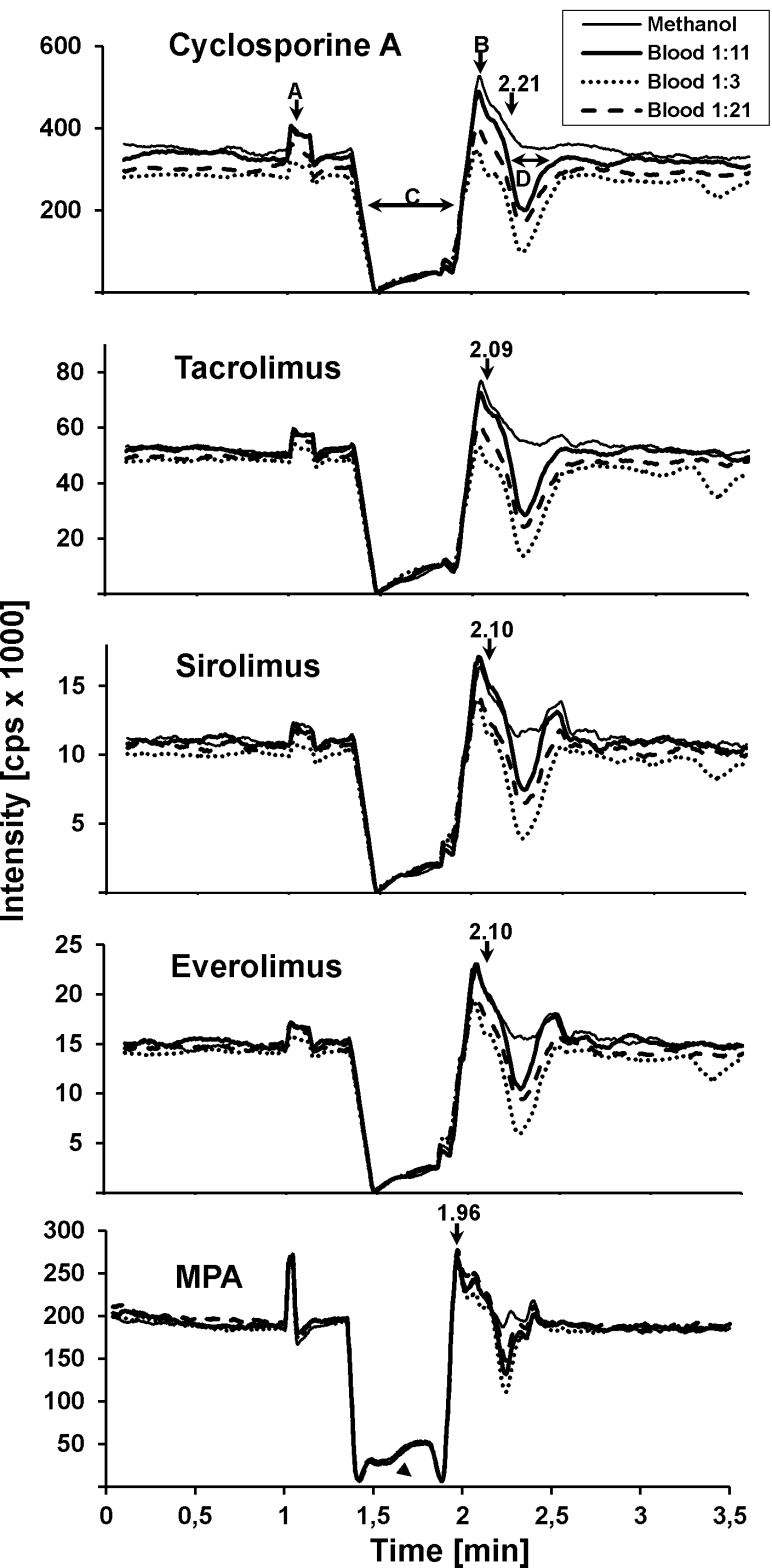
**Monitoring ion suppression (post column infusion method)**. The TIC of all immuno-suppressants when injected in methanolic solution, 1:3, 1:11 and 1:21 diluted sample-matrix respectively are shown. **A**: valve switch to methanolic buffer (97% methanol). **B**: valve switch to aqueous buffer (10% methanol; reconditioning of SPE column). **C**: strong loss of ions during straight forward elution of aqueous buffer from SPE column. **D**: second loss of signal during assumed elution of sample matrix (2.15 - 2.45 min).

Figure 4 depicts the monitoring of matrix effects by means of post column infusion in conjunction with the corresponding retention times. The graphical time course of the total ion count is illustrated by the CSA figure. After the valve switch to methanolic buffer (A), the sample was flushed to the analytical column. Strong ion loss occurred during the following elution of aqueous buffer from the SPE column to the analytical column (C). Reconditioning of the SPE column (B) started at 1.9 min, when the valve switched back to position A.

A second signal loss appeared during the assumed elution of sample matrix (D).

Compared to methanol-based solutions, the three tested dilutions of sample preparation exhibit distinct matrix effects (Figure 4). As expected, these effects are more pronounced at a lower sample dilution (1:3). Surprisingly, the higher dilution of 1:21 resulted in stronger matrix effects than the lower dilution of 1:11. Due to a lack of significant reduction of matrix effects and increased imprecision at higher dilutions, the 1:11 ratio was chosen for further development of this application.

The time frame of ion suppression ranged from 2.15 to 2.5 min. With 2.21 min., the retention time of CSA was within this time window, whereas TAC, SIR and EVE were not affected (retention time: 2.10 min.). As illustrated in terms of quality, CSA showed a high and TAC a slight ion suppression, whereas SIR, EVE and in MPA exhibited an ion enhancement. These effects were offset by the deuterated internal standards.

**Figure 5:**
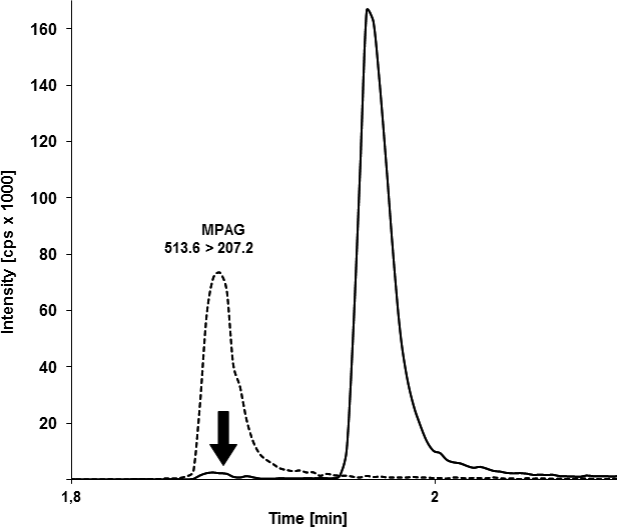
**Chromatographic separation of MPAG m/z 513.6/207.2 (dotted line) and MPA m/z 338.2/207.1 (solid line)**. The arrow indicates the in-source fragmentation of MPAG to MPA.

Figure 5 depicts the distinct chromatographic separation of MPAG and MPA. The in-source fragmentation of MPAG to MPA is shown by the signal of the m/z 338.1/207.1 trace within the MPAG peak. Concerning MPA, comparative measurements of mass spectrometry and immunoassay showed significantly different results for samples with a high content of MPAG (data not shown). This discrepancy of up to 50% reflects, among other things, a high cross-reactivity of the antibody against the glucuronide.

Sample stability, as confirmed by repeated measurements of 15 extracted patient samples per analyte after 5 hours, resulted in a CV < 8%.

Participation in the international proficiency testing scheme in 2010 (analytical services international ASI, Prof. Holt, U.K) revealed a mean accuracy of 3.6% for CSA, 10.2% for TAC, -3.5% for SIR, 2.1% for EVE and -2.6% for MPA and confirmed the validity of the method.

## Discussion

When determining immunosuppressants in whole blood, thorough sample preparation plays a crucial role. Complete lysis of erythrocytes and protein precipitation are mandatory for good reproducibility. We employed water incubation in conjunction with ultrasound treatment to ensure complete lysis, which was microscopically verified at the beginning of the validation process and ensured high reproducibility of the measurements. Water lysis alone left many erythrocytes undamaged and compromised precision.

Second, internal standards play a key role in mass-spectrometric analyses. The use of CSD, ascomycin and MPAC as internal standards for the quantification of immunosuppressants can cause measurement errors due to an ionization process in the ESI source which deviates from that of the analyte [6,7,20-22]. Initially, the common standards were also used for this validation protocol. However, especially for CSA, divergence of ionization efficiency (analyte vs. IS) were so large that the results were significantly distorted.

This effect became particularly evident in matrices of two certified control materials. In this case, CSA was subject to ion enhancement, resulting in 40% higher values than the specified target. The use of deuterated standards could compensate for these matrix effects and resulted in correct recovery. One batch of calibrators was also affected by this phenomenon. In routine clinical measurements, matrix impacts based on (non-standardized) patient samples will always be a recurrent problem, which could be overcome by the use of deuterated standards.

Crucial for correct recovery is an IS which comes chemically as close as possible to the analyte. The close chemical relationship leads to identical retention time, so that IS and analyte are affected in the same way, and matrix effects can be compensated for.

Ascomycin also exhibited an elution behavior dependent on the sample matrix, making it liable to wide fluctuations. By use of deuterated standards that eluted concurrently with the analytes, we noted that measurement errors, especially those caused by ion suppression or enhancement, were nearly all compensated for.

In contrast to other laboratories, the working solution for sample preparation was set up daily to avoid any instability. Small sample batches contributed to a fast processing time, which could be kept below one hour. Repeated analyses of samples after five hours confirmed the previous results and thus verified the stability of the extracted samples.

The analysis time required for this method is 3.5 minutes. We are highly suspicious of attempts to shorten analysis times to one minute [8], especially when dealing with substances having a variety of metabolites like CSA, as isobars may appear which interfere with the analyte, skewing the results. The likelihood of such interferences can be minimized by thorough HPLC separation.

One issue in developing this application was the identification of the most appropriate sample dilution. The initial dilution of 1:3 was raised to 1:21 so as to reduce matrix effects and soiling of the equipment [17]. Later, the dilution was reduced to 1:11 due to the observation that higher dilutions failed in further reduction of matrix effects and lead rather to greater imprecision, in spite of the equipment's sensitivity.

Precision was not only influenced by dilution but also depended on the material used. Certified lyophilized control material used for the determination of LOQ revealed a lower CV than spiked patient's blood for intra- and inter-assay testing. This phenomenon may possibly be explained by the different matrices.

Another factor of influence was the purity of the reagents. Many annoying adducts became apparent in water and methanol when using HPLC-grade reagents. These adducts have considerably reduced the sensitivity.

As was noted by Annesley [23], we also observed significant differences in signal strengths from the same sample, even when using several LC/MS grades of methanol from various companies.

Most laboratories employ an HPLC-UV method or immunoassay to determine MPA. Our method herein stands out not just because CSA, TAC, SIR and EVE can all be measured in parallel, but because MPA can also be quantified using the same application. However, due to the fact that no commercial calibrator is available containing MPA, it is inevitable that a separate calibration is necessary. The additional expenditure is compensated by identical analytical conditions and procedures for all analytes. In agreement with results from other working groups, we failed to determine MPA in whole blood due to inadequate precision and accuracy (personal communication). Currently there is no explanation for this result. As a consequence, sample preparation must be carried out with plasma. However, additional UV-HPLC analysis for the determination of MPA, as is customary in most laboratories, can be waived.

As mentioned above, the chromatographic separation of MPAG and MPA is crucial due to in-source fragmentation of MPAG to MPA, which is then added to the intrinsic value of MPA in the case of coelution [11]. The extent to which this secession occurs is essentially construct-dependent. Glucuronide splits off to such a miniscule degree on our equipment that we could forgo chromatographic separation, although for reasons of safety, we do not. Different retention times for MPA and MPAG could be realized by a flow-through procedure on the online-SPE column.

Adequate separation of MPA from MPAG proved impossible when we tried to shorten the analysis time to 2.5 minutes or using the back flush online SPE method.

We compared our method to LC-MS/MS methods used by two university and commercial laboratories, observing that our results differed strikingly from the latter. This clearly illustrates that it is not just the immunoassays which need to be standardized - the physical methods must be standardized as well in order to guarantee comparability.

The wide divergence of LC-MS methods is reflected in the international proficiency testing scheme's evaluation and is caused by differences in equipment, applications, and sample preparation. Furthermore, this application is regularly confirmed via national proficiency testing (DGKL, Instand).

Surprisingly, the signal strengths of the analytes and the IS differ significantly even when identically-constructed instruments from the same manufacturer are used, because of the sources' divergent ionization behavior. By changing the heating units and probes we observed an up to a ten-fold difference in signal strengths when comparing three sources on two mass spectrometers. At this point, the manufacturer has a duty to develop better standardization procedures in order to improve quality control. Devices should not be tested with a reference source, but with the supplied sources.

The prevalence of immunosuppressive combination therapy allows dose reduction and minimizes undesirable side effects [2,24]. However, it makes greater demands on the analysis systems used, especially concerning precision and reproducibility [19]. This explains why replacement of immunoassays by mass spectrometry is taking root - it offers significantly better sensitivity and is highly specific for the drug's parent compound, without having cross reactivity to metabolites. In the case of CSA, the average recovery of immunoassays is up to 25% higher compared to mass spectrometry. This difference is due to individually occurring metabolites with unknown pharmacologic effects.

In addition, economic considerations also favor the mass-spectrometry based method. First, it is time saving since a single analytical run is adequate for the determination of CSA, TAC, SIR and/or EVE in patients with combination therapy. Although two analytical runs are needed in the case of MPA co-administration, the saving in time is still evident. Second, the authors were able to reduce the reagent and consumable costs by more than 80% compared to the previously used immunoassays (340,000 Euro/year for CSA, TAC and MPA vs. 50,000 Euro/year for all analytes).

Arguments against mass spectrometry are its high acquisition costs, high skill level required of personnel, and the complex validation process necessary, so that academic expertise is indispensable. Moreover, in the case of defects or maintenance, the laboratory may require a back-up system.

But nevertheless, once the mass-spectrometric method has been established, it is easy to use in daily routine. Therefore, the authors recommend this method for laboratories with high throughput such as university hospitals with transplantation departments.

## Conclusion

Before mass spectrometric applications can be employed for routine clinical purposes or be considered to be a gold standard, they must be subjected to a thorough validation process. The use of deuterated internal standards is highly recommended since the commonly used standards may cause analytical problems due to an ionization efficiency differing from that of the analyte.

Last but not least: without established standards the findings from different laboratories cannot be reliably compared.

## Competing interests

The authors declare that they have no competing interests.

## Authors' contributions

AB and TE were responsible for the development of sample preparation, chromatography conditions and mass-spectrometer settings. KW participated in the statistical analysis of the results. All authors contributed to the content and review of the manuscript and read and approved the final manuscript.

## Pre-publication history

The pre-publication history for this paper can be accessed here:

http://www.biomedcentral.com/1472-6904/12/2/prepub
